# Magnetic Resonance Spectroscopy of 2‐Hydroxyglutarate and Glycine in Adult Subjects With Brainstem Gliomas

**DOI:** 10.1002/nbm.70153

**Published:** 2025-09-30

**Authors:** Vivek Tiwari, Sandeep K. Ganji, Zhongxu An, Marco C. Pinho, Larry T. Davis, Colin D. McKnight, Julia D. Berry, Bret C. Mobley, Leo Y. Luo, Alexander C. Mohler, Ryan T. Merrell, Patrick D. Kelly, Reid C. Thompson, Bruce E. Mickey, Craig R. Malloy, John C. Gore, Toral R. Patel, Elizabeth A. Maher, Changho Choi

**Affiliations:** ^1^ Department of Biological Sciences Indian Institute of Science Education and Research Behampur Odisha India; ^2^ Philips Cambridge Massachusetts USA; ^3^ Department of Radiology Mayo Clinic College of Medicine Rochester Minnesota USA; ^4^ Advanced Imaging Research Center University of Texas Southwestern Medical Center Dallas Texas USA; ^5^ Department of Radiology University of Texas Southwestern Medical Center Dallas Texas USA; ^6^ Department of Radiology and Radiological Sciences Vanderbilt University Medical Center Nashville Tennessee USA; ^7^ Department of Pathology, Microbiology and Immunology Vanderbilt University Medical Center Nashville Tennessee USA; ^8^ Department of Radiation Oncology Vanderbilt University Medical Center Nashville Tennessee USA; ^9^ Department of Neurology Vanderbilt University Medical Center Nashville Tennessee USA; ^10^ Department of Neurological Surgery Vanderbilt University Medical Center Nashville Tennessee USA; ^11^ Department of Neurological Surgery University of Texas Southwestern Medical Center Dallas Texas USA; ^12^ Department of Internal Medicine University of Texas Southwestern Medical Center Dallas Texas USA; ^13^ Vanderbilt University Institute of Imaging Science Vanderbilt University Medical Center Nashville Tennessee USA; ^14^ Department of Biomedical Engineering Vanderbilt University Nashville Tennessee USA; ^15^ Department of Physics and Astronomy Vanderbilt University Nashville Tennessee USA; ^16^ Department of Neurology and Neurotherapeutics University of Texas Southwestern Medical Center Dallas Texas USA; ^17^ Department of Radiation Oncology University of Texas Southwestern Medical Center Dallas Texas USA; ^18^ Department of Psychiatry and Behavioral Sciences Vanderbilt University Medical Center Nashville Tennessee USA

**Keywords:** 2‐hydroxyglutarate, 3T, brainstem tumor, gliomas, glycine, magnetic resonance spectroscopy, PRESS (point‐resolved spectroscopy)

## Abstract

Surgical biopsy of brainstem tumors carries a risk of neurological injury. We performed magnetic resonance spectroscopy (MRS) of 2‐hydroxyglutarate (2HG) and glycine in patients with brainstem tumors to assess the feasibility of detecting and quantifying 2HG in the brainstem to obviate the need for a diagnostic biopsy and to establish the clinical significance of glycine MRS in brainstem tumors in vivo. Twenty adult patients with radiographically identified presumed brainstem gliomas were prospectively enrolled in the study. Proton MRS was obtained at 3T with a protocol tailored for detection of 2HG and glycine (TE 97‐ms PRESS). Spectra were fit using LCModel software and in‐house basis signals of metabolites and lipids. The metabolite concentrations were quantified with reference to water and examined with respect to clinical outcomes, including postgadolinium MRI and overall survival time. MRS data from 19 patients were included in subsequent analysis, excluding suboptimal data from one patient. Tumors with elevated 2HG (> 1.9 mM, *N* = 8) and undetectable 2HG (< 0.3 mM, *N* = 11) were clearly distinguishable. Tumors with elevated glycine (> 1.5 mM, *N* = 4) showed rapid progression. Kaplan–Meier survival analyses with metabolite measures demonstrated that tumors with 2HG higher than 1.0 mM were significantly associated with a favorable prognosis (*p* = 0.01). In contrast, tumors with glycine higher than 2.5 mM showed a strong association with poor survival (*p* = 0.0005). The data confirm detection of 2HG in brainstem tumors at a concentration that is consistent with an IDH mutation and expected good prognosis, whereas elevated glycine in brainstem tumors portends rapid tumor progression and a worse prognosis.

## Graphical abstract

MRS of 2‐hydroxyglutarate (2HG) and glycine in 19 patients with brainstem tumors is reported. TE 97ms PRESS was obtained at 3T and LCModel was used for spectral fitting. Tumors with elevated 2HG and undetectable 2HG were clearly distinguishable. Tumors with elevated glycine showed rapid progression. Kaplan‐Meier analyses demonstrated that tumors with 2HG higher than 1.0 mM were significantly associated with favorable prognosis. In contrast, tumors with glycine higher than 2.5 mM showed strong association with poor survival.

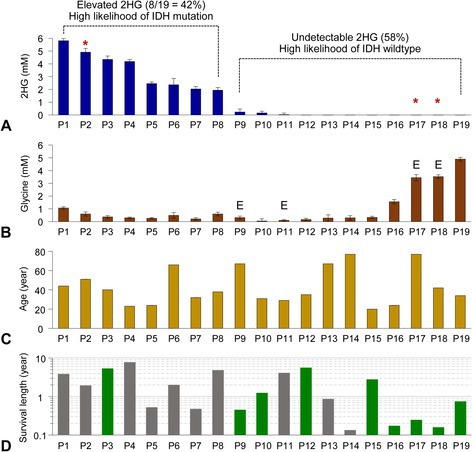

**Abbreviations**
2HG: 2‐hydroxyglutarate
CRLB: Cramer–Rao lower bounds
FWHM: full width at half magnitude
IDH: isocitrate dehydrogenase
PRESS: point‐resolved spectroscopy
RF: radio frequency
SD: standard deviation
SNR: signal‐to‐noise ratio
STEAM: stimulated‐echo acquisition mode
T1‐Gd: T1‐weighted MRI postgadolinium
T2‐FLAIR: T2‐weighted fluid attenuated inversion recovery MRI
tCho: total choline (= glycerophosphocholine + phosphocholine)
tCr: total creatine (= creatine + phosphocreatine)
tNAA: total NAA (= N‐acetylaspartate + N‐acetylaspartylglutamate)

## 1 Introduction

Brainstem tumors are rare in adults [1, 2]. The nuclei and white matter tracts within the brainstem underlie the basic vital functions of the human body. Thus, surgical biopsy of brainstem lesions may carry a high risk of morbidity. The diagnosis of a brainstem lesion therefore usually relies on radiological features. However, in the absence of a tissue sample, the clinical management of brainstem tumors cannot account for varying tumor genotype and alterations in metabolism.

A subset of gliomas harbors mutations in isocitrate dehydrogenase (IDH) and produces D‐2‐hydroxyglutarate (2HG) [3]. As a result, 2HG, which is normally present in only micromolar levels, is increased to millimolar levels [3]. IDH‐mutated gliomas have a more favorable prognosis than IDH‐wildtype tumors [4, 5], so detection of elevated 2HG by MRS may be predictive of longer survival. Numerous 2HG MRS studies have been reported since the earliest detection of 2HG by MRS [6, 7, 8].

The FDA approval of the IDH inhibitor, Vorasidenib, in Grade 2 gliomas harboring an IDH1 or 2 mutation based on the statistically and clinically significant randomized Phase 3 clinical trial data demonstrating prolonged progression‐free survival and time to next intervention [9], has raised the question of whether this drug could be a useful therapy in low‐grade brainstem gliomas. Due to the high risk of neurological injury from biopsy of the brainstem, patients with presumed brainstem gliomas have been treated with early radiation, which carries high morbidity. In brainstem tumors that may harbor an IDH mutation and be candidates for the IDH inhibitor, tissue immunohistochemistry or genetic sequencing is required for commercial or patient assistance approval.

Tumors also reprogram metabolism to enhance normal metabolic production of amino acids and nucleotides via one‐carbon metabolism [10, 11, 12]. The rates of glycine consumption and expression of glycine biosynthetic enzymes may be increased in highly proliferating cancer cells [13, 14]. Ex vivo [15, 16] and in vivo [17, 18, 19, 20, 21, 22] MRS studies have shown elevations of glycine in high‐grade brain tumors. In a recent MRS study of adult patients with supratentorial gliomas, glycine levels were positively correlated with cellular proliferation rates, and tumors with high glycine were significantly associated with poor survival [23].

Point‐resolved spectroscopy (PRESS) is a standard MRS acquisition technique widely used in clinical studies at 3T. Although short echo‐time (TE) MRS benefits from minimal T2 signal loss, a long‐TE approach can improve the detection of metabolites, with the advantage that spectral analysis is simplified with the attenuation of complex macromolecular signals. Moreover, TE can be tailored to improve the detection of specific target metabolites. For instance, TE 97‐ms PRESS has proven to be optimal for detecting 2HG and glycine in the human brain [6, 23, 24].

Given the difficulties with surgical biopsy of the brainstem, the ability to noninvasively evaluate molecular alterations in brainstem tumors is important for clinical management. Here, we present a preliminary assessment of the clinical utility of metabolite measurements in brainstem tumors, as measured with TE 97‐ms PRESS. MRS measures of 2HG, glycine, and other metabolites were collected to examine their relationships to tumor aggressiveness and patient survival.

## 2 Methods

### 2.1 Subject Enrollment

Twenty adult patients with radiographically identified presumed brainstem gliomas were prospectively enrolled in IRB approved studies at two sites: fourteen subjects at University of Texas Southwestern Medical Center between 2014 and 2021 and six subjects at Vanderbilt University Medical Center between 2022 and 2024. Aside from a subject whose data were excluded due to inadequate quality (low signal‐to‐noise ratio [SNR]) (Table S1), the age of 19 subjects ranged from 20 to 77 years (mean 43 ± 19 years) at the time of the first MRS scan. Fifteen subjects had MRS at a single time point and four participants at multiple time points. There was no treatment prior to the first MRS exam in all patients. Of the four patients with multi‐MRS exams, two patients underwent chemoradiation therapy between the first and second MRS exams, and two other patients had no treatments between the first and follow‐up MRS scans. Tumor biopsy was clinically analyzed for IDH mutational status and histological tumor grading in three patients. IDH status was determined by immunohistochemistry, followed by DNA sequencing when necessary. Five healthy adult volunteers (age 26–38 years) were enrolled for control MRS in brainstem, medial posterior, and left parietal brain. The human MR protocols were approved by the local Institutional Review Boards. Written informed consent was obtained from each patient prior to the MR scans.

### 2.2 MR Data Acquisition

^1^H MRI and single‐voxel localized MRS data were acquired using research‐dedicated Philips 3T scanners, equipped with whole‐body transmit coils and 32‐channel receive head coils. T2‐FLAIR images were acquired and used for MRS voxel positioning together with postgadolinium T1 weighted images that were acquired in clinical scans 24 h before the MRS at minimum. Care was taken to avoid cyst and/or highly edematous volumes in the voxel positioning. A water‐suppressed PRESS spectrum was acquired, with TE 97‐ms PRESS (*TE*_1_ = 32 ms and *TE*_2_ = 65 ms) [6, 24], from a voxel positioned within the T2‐FLAIR hyperintense and gadolinium enhancing volume, when applicable. The voxel size ranged from 1.5 to 8 mL, for which the number of MRS signals averaged was set to range from 896 to 128 (Figures S1 and S2A) to achieve similar SNR across the voxel size (Figure S2B). Water‐suppressed time‐domain signals were recorded in multiple blocks, each with 16 RF phase cycled signal averaging. The PRESS scan parameters included 2‐s repetition time, 2.5‐kHz spectral width, and 2048 sampling points. The PRESS sequence had vendor‐supplied radio‐frequency (RF) pulses (sharp): 9.8 ms 90° pulse (bandwidth 4.2 kHz) and 13.2 ms 180° pulses (bandwidth 1.3 kHz), all at an RF field intensity (*B*_1_) of 13.5 μT. The carrier frequencies of the PRESS RF pulses were set at 2.7 ppm. Water suppression was obtained using four variable flip‐angle pulses [25]. *B*_0_ shimming was performed up to second order with a vendor‐supplied tool. Flip angle calibration was conducted on the *B*_0_ shimming volume (21–27 mL). Frequency drifts were corrected in real time for each excitation using a vendor‐supplied tool (Frequency stabilization). Unsuppressed water signal was acquired with the PRESS TE 97‐ms sequence for use as reference in multichannel combination and eddy current compensation. In addition, unsuppressed water spectra were recorded at TE of 14 ms using a stimulated‐echo acquisition mode (STEAM) sequence and TR of 20 s for use as a reference in metabolite quantification. This long‐TR water and the PRESS acquisitions were preceded by a TR 2‐s water scan, which was undertaken to ensure acceptable *B*_0_ shimming. When necessary, the TR 2‐s water was repeatedly acquired after adjusting the size and/or orientation of the *B*_0_ shimming volume and/or the MRS voxel (up to 3–5 min). The total scan duration was 10–40 min, including water acquisitions, *B*_0_ shimming, and flip‐angle calibrations. The Philips scanner software version used included R5.3, R5.7, and R5.9 over the study period. There was no notable difference between the versions in terms of MRS data quality. Additional information is shown in Table S1.

### 2.3 MR Data Processing

Multichannel data combination and eddy current compensation were performed with vendor‐supplied algorithms. The multiblock single‐voxel MR data were corrected for frequency drifts with reference to a prominent singlet prior to final signal averaging. Here, the reference peak was the N‐acetylaspartate singlet for healthy brain data and the choline singlet for tumor data [24, 26]. Following apodization with a 1‐Hz exponential function, spectral fitting was performed with LCModel software (Version 6.3‐1L) [27], using in‐house basis spectra of metabolites that were numerically calculated including the effects of the PRESS slice selective RF and gradient pulses [6]. The basis set comprised model spectra of 23 metabolites, which included 2HG, glycine, glutamate, glutamine, GABA, myo‐inositol, lactate, citrate, glutathione, alanine, aspartate, ethanolamine, phosphoethanolamine, scyllo‐inositol, taurine, glucose, ascorbate, glycerophosphocholine, phosphocholine, creatine, phosphocreatine, N‐acetylaspartate, and N‐acetylaspartylglutamate. Two customized basis signals of lipids, created with the LCModel built‐in algorithm according to a prior study [26], were included in the fitting. The spectral fitting was performed between 0.5 and 4.0 ppm (sptype = “tumor” and default baseline spline option), excluding the LCModel default basis signals of lipids and macromolecules, similarly to a prior study [26]. The metabolite signal estimates were normalized to the STEAM water signal multiplied by 2, and subsequently, the millimolar concentrations of metabolites in healthy brain and brainstem tumors were calculated with reference to water at 40 and 48 M, respectively, similar to a prior study [26]. Spectra having sufficiently small background noise levels (i.e., small residuals) were included in subsequent analyses (Figure S1).

### 2.4 Statistical Analysis

The percentage Cramer‐Rao lower bounds (CRLB) of metabolite signal estimates were returned by LCModel as the precision of the signal estimates. Unpaired *t* test was performed for group comparison of metabolite estimates. The *p* values from the *t* test were corrected for false discovery rates using the Benjamini–Hochberg method [28], which gave *q* values. Kaplan–Meier analysis of metabolite measures was performed to compare overall patient survival. Hazard ratios were estimated with Cox regression models. The survival time was defined as the interval from the MRS scan date to the date of death. For each metabolite, a cutoff value that divided the estimates into two groups with the smallest 95% confidence interval was determined for the survival analysis. All data are presented as mean ± standard deviation (SD). Statistical significance was declared at *p* or *q* ≤ 0.05.

## 3 Results

**FIGURE 1:**
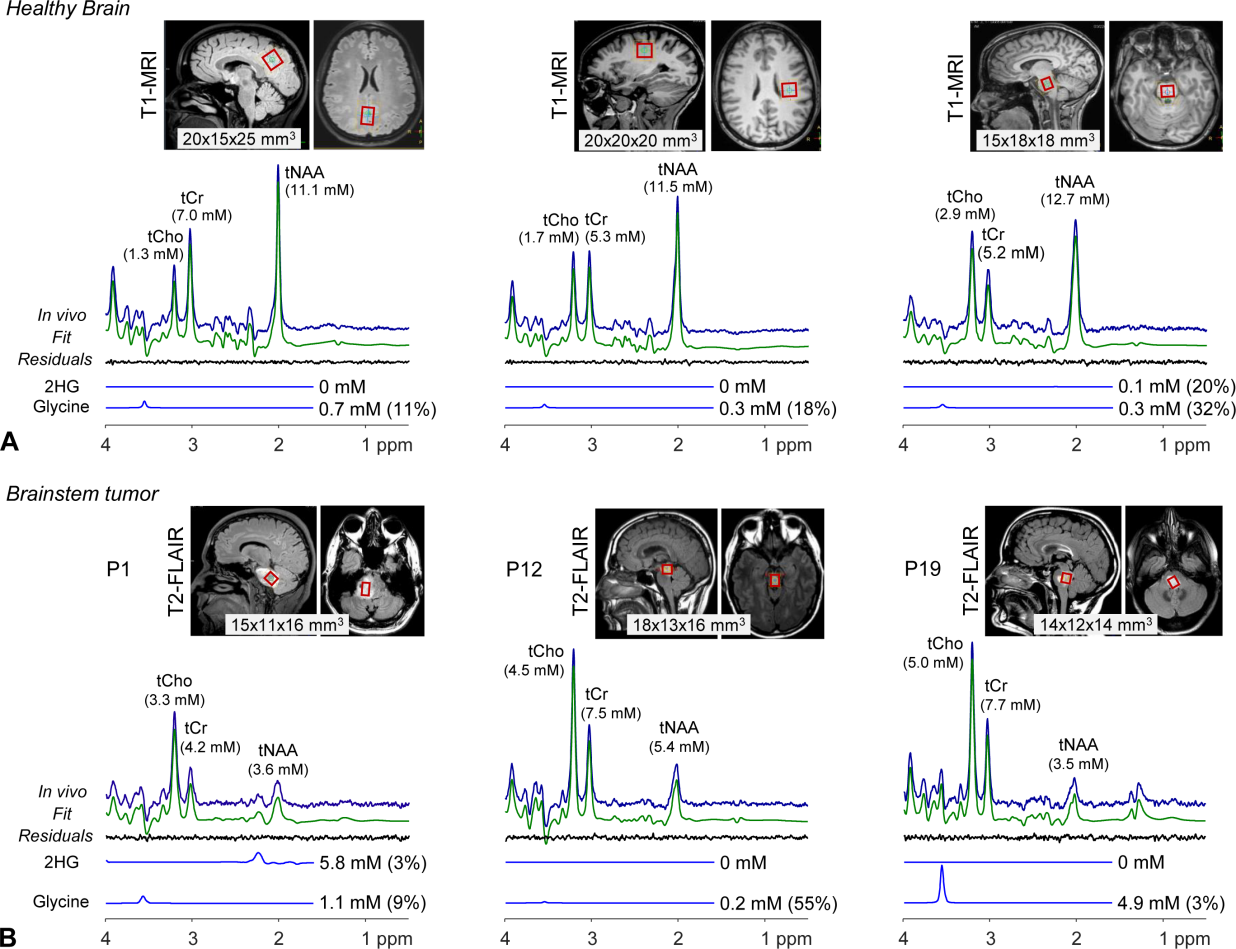
Representative in vivo ^1^H MR spectra (A) from the medial posterior and left parietal regions and brainstem in healthy brain and (B) from brainstem lesions in three patients (P1, 44‐year‐old male; P12, 35‐year‐old male; and P19, 34‐year‐old female) are presented together with spectral analyses and voxel positioning (red lines) on T2‐FLAIR images (voxel size shown within the images). LCModel‐returned 2HG and glycine signals are shown with the millimolar concentration estimates and CRLB values in brackets. Spectra are scaled with respect to the water signals from the voxels. The lesions in the three patients were nonenhancing at the time of MRS.

In healthy brain, the composite pattern of tCho (glycerophosphocholine + phosphocholine), tCr (creatine + phosphocreatine), and tNAA (N‐acetylaspartate + N‐acetylaspartylglutamate) signals was notably different between the brainstem and medial posterior and left parietal brain (Figure 1A). For the five subjects of the present study, although the tNAA level was similar between the three regions (11.5 ± 0.7, 11.7 ± 0.9, and 12.9 ± 0.7 mM for medial posterior, left parietal, and brainstem, respectively), the tCho/tCr concentration ratio was markedly higher in the brainstem compared with the two other brain regions (0.56 ± 0.04 vs. 0.24 ± 0.06; *n* = 5 and 10; *q* = 2 × 10^−7^). The tCho level was twofold higher in the brainstem than in the two other regions (3.0 ± 0.2 vs. 1.5 ± 0.2 mM; q = 2 × 10^−8^). 2HG was undetectable in the healthy brain. Glycine was estimated at 0.3–0.7 mM in the three brain regions. The brainstem 2HG and glycine estimates in the five healthy subjects were 0.1 ± 0.1 and 0.4 ± 0.1 mM, respectively.

For brainstem tumors, the pattern of high tCho and low tCr and tNAA signals was seen in all cases (Figure 1B). Compared to normal brainstem, although tNAA estimates in brainstem tumors were much lower, the tCho/tCr ratios in tumors were not notably higher. In a spectrum from a patient with a nonenhancing tumor (P1), a large signal was clearly discernible at 2.25 ppm, corresponding to a high concentration of 2HG (5.8 mM). The glycine plus myo‐inositol composite signal pattern between 3.5 and 3.7 ppm in the tumor was different from those in healthy brain, indicating an increased glycine signal at 3.55 ppm. The glycine level was estimated as 1.1 mM, twofold to threefold higher than the normal brainstem glycine level. In a patient with a nonenhancing pontine tumor (P12), 2HG was not measurable, and the glycine estimate was low (0.2 mM). In a patient with a nonenhancing midbrain lesion (P19), 2HG was not measurable, and the glycine level was very high. Spectral analysis returned 4.9‐mM glycine, approximately 12‐fold higher than the normal brainstem glycine level. The 2HG and glycine signals and adjacent resonances of other metabolites were acceptably resolved from each other in the normal‐brain and brain‐tumor spectra (Figure S3).

For the 19 tumor patients, the mean FWHM of LCModel‐returned tCho signal was 7.3 ± 1.5 Hz (range 5.2–10.3 Hz) after 1‐Hz exponential apodization (Figure S1). The tCho SNR was 97 ± 29 (range 38–157) (Figure S2B). Here, the SNR was the ratio of the tCho singlet amplitude relative to the SD of the LCModel‐returned residuals between 0.2 and 4.0 ppm.

**FIGURE 2:**
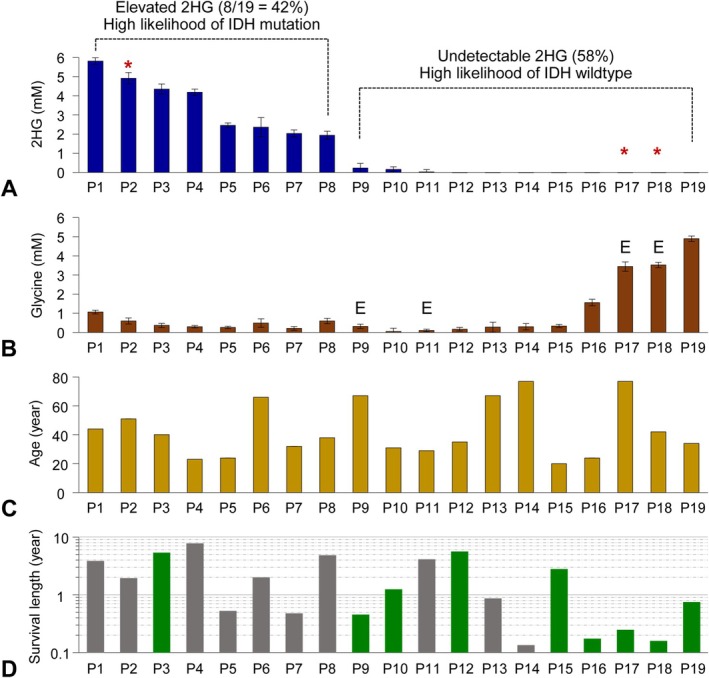
(A, B) The millimolar concentration estimates of 2HG and glycine in 19 patients (P1–P19) are bar graphed in the descending order of 2HG followed by the ascending order of glycine for null 2HG cases. The 2HG estimates in eight patients (P1–P8) ranged from 1.9 to 5.8 mM and those in other 11 patients (P9–P19) were less than 0.3 mM (zero 2HG in P12–P19). The glycine estimates in the 19 patients ranged from 0.1 to 4.9 mM. Tumor tissue was obtained in three patients (indicated by red asterisks) and the analysis of the tissue confirmed MRS prediction of the IDH mutational status (i.e., IDH‐mutated WHO Grade 3 astrocytoma in P2 and IDH‐wildtype glioblastomas in P17 and P18). Patients with enhancing lesions at the time of the first MRS are labeled with E (P9, P11, P17, and P18). The error bars represent standard deviations calculated from LCModel‐returned CRLBs. (C) The ages (years) of the 19 patients at the time of the first MRS exam. (D) The survival times of nine patients with a documented date of death are bar graphed (green) with a log scale of years. The bars in gray indicate the time between MRS and end of the study.

The 2HG estimates ranged from 0 to 5.8 mM (Figure 2A). Two groups of patients were clearly identifiable: patients with elevated 2HG (P1–P8 group) and those with undetectable 2HG (P9–P19 group). Based on our prior MRS studies validating IDH mutations in resected tumors, 2HG > 1.0 mM was highly correlated with IDH mutation, whereas 2HG < 1.0 mM was either IDH wildtype tumor or low‐cellularity IDH‐mutant tumor [26, 29]. The 2HG estimates in the eight patients with elevated 2HG ranged from 1.9 to 5.8 mM, consistent with the diagnosis of IDH‐mutant gliomas in resected tumors. The 2HG estimates of the P9–P19 tumors ranged from 0 to 0.3 mM, most consistent with a lack of IDH mutation (IDH wildtype) or possibly with a low‐cellularity mutant‐IDH tumor. The glycine estimates in the 19 patients ranged from 0 to 4.9 mM (Figure 2B). The mean glycine concentration was about threefold higher in the undetectable‐2HG tumors than in the elevated‐2HG tumors (1.4 ± 1.7 vs. 0.5 ± 0.2 mM) but did not reach statistical significance (*p* = 0.17). Four patients with high glycine levels (P16–P19) had a short median overall survival (< 1 year) (Figure 2C), consistent with our prior MRS study showing high glycine being correlated with high‐grade disease [23]. The mean tCho/tCr concentration ratio in brainstem tumors was not significantly higher compared to normal brainstem (0.81 ± 0.28 vs. 0.58 ± 0.03; *N* = 19 and 5, respectively; *p* = 0.06).

**FIGURE 3:**
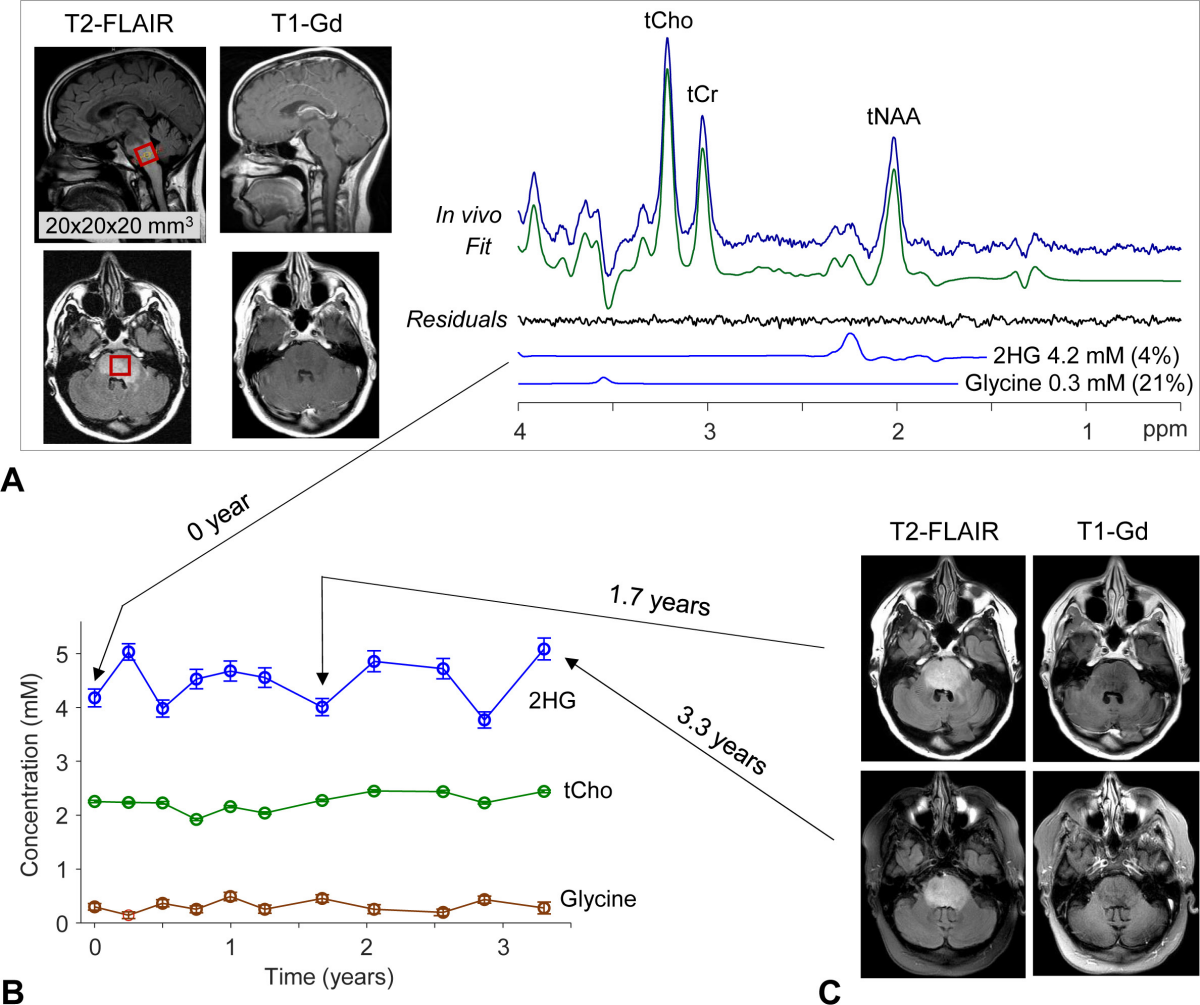
Longitudinal MRS in a brainstem tumor patient (P4). (A) The spectrum of the first MRS scan is presented together with spectral analysis results, voxel positioning on T2‐FLAIR images, and postgadolinium T1 MRI. The LCModel‐returned 2HG and glycine signals are shown together with the concentration estimates and CRLB values in brackets. (B) The estimates of 2HG, tCho (total choline), and glycine in 11 MRS exams in the patient, obtained over a 3.3‐year period, are plotted versus time. The mean concentration estimate over the study period was 4.5 ± 0.5 mM for 2HG, 2.2 ± 0.2 mM for tCho, and 0.3 ± 0.1 mM for glycine. The error bars represent the standard deviation calculated from CRLBs. (C) During the entire study period, the T2‐FLAIR volume remained about the same, and the tumor stayed nonenhancing, as illustrated by the T2‐FLAIR and postcontrast T1‐MRI at 1.7 and 3.3 years from the enrollment.

We had the opportunity to perform prolonged serial MRS in a patient with a nonenhancing pons‐medulla lesion (P4) who had presumed low‐grade disease at presentation and an initial MRS showing detectable 2HG (4.2 mM) (Figure 3A) and normal levels of glycine. The patient was followed off treatment with the intent to delay radiation. The tumor was stable radiographically without enhancement and had stable 2HG measurements on 11 MRS exams over 3.3 years (3.8–5.0 mM), consistent with the presumptive diagnosis of an IDH‐mutant low‐grade glioma. Glycine remained low and tCho was unchanged, consistent with stable low‐grade disease (Figure 3B,C). The metabolic profile was also stable during the study period (Figure S4). This dataset provided unique longitudinal follow‐up of the imaging biomarkers using the stable clinical imaging over > 3 years as a validation tool.

**FIGURE 4:**
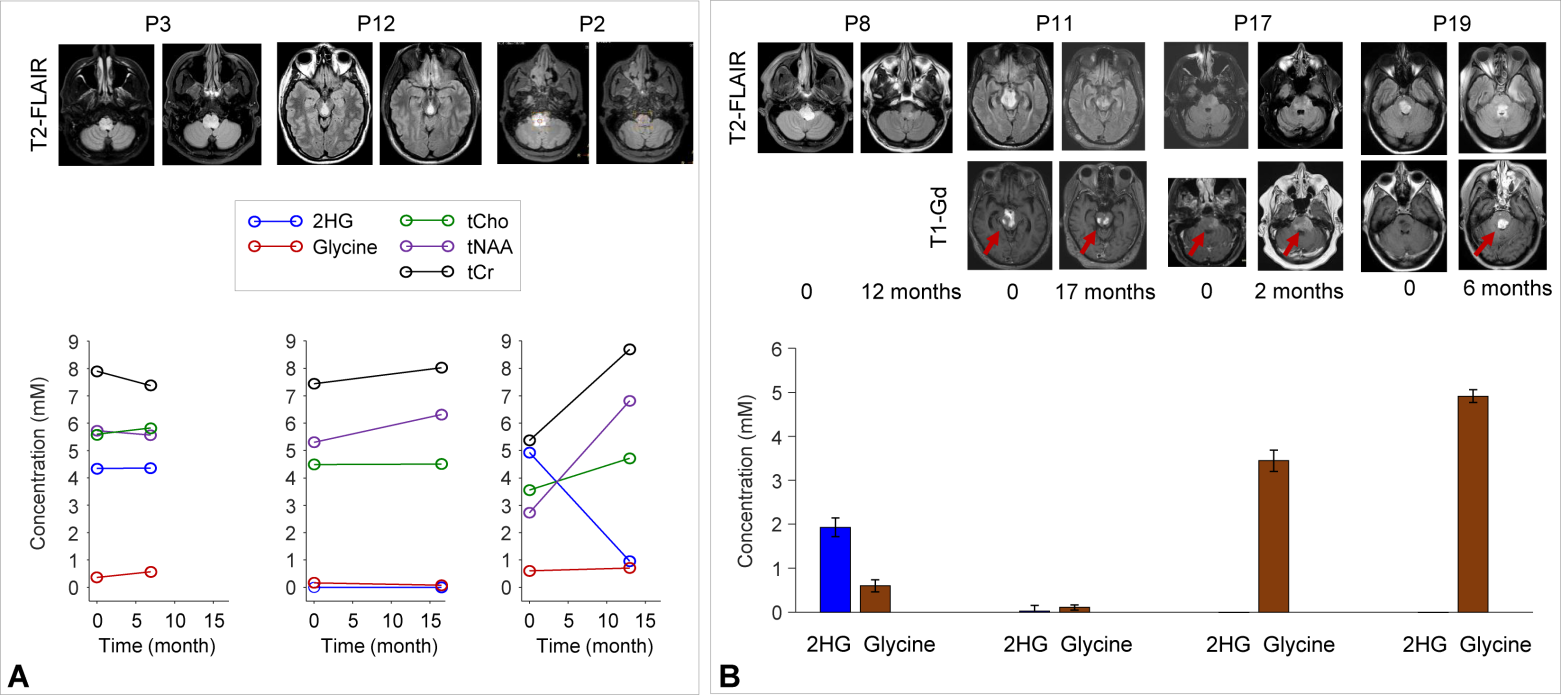
(A) Metabolite estimates in three patients (P3, 40‐year‐old male; P12, 35‐year‐old male; and P2, 51‐year‐old male), who had MRS at two time points, are presented together with T2‐FLAIR. For each patient, the MR images on the left and right correspond to the first and second MRS time points, respectively. The brainstem lesions were nonenhancing during the MRS study period. (B) For four patients (P8, 38‐year‐old male; P11, 29‐year‐old male; P17, 77‐year‐old male; and P19, 34‐year‐old female), T2‐FLAIR and T1‐Gd images at two time points are presented together with 2HG and glycine estimates at the first MRI time point (0 month). The lesion in P8 was nonenhancing during the study period.

Three patients had MRS scans at two time points (Figure 4A). A patient with a stable nonenhancing right midbrain tumor (P3) had an initial 2HG level of 4.3 and 4.4 mM on the follow‐up scan 7 months later without treatment. Similarly, glycine was low in both MRS exams (0.4–0.6 mM). Reflecting differences in treatment approaches in presumptive brainstem gliomas without enhancement (likely low‐grade), the second and third patients both underwent chemotherapy and radiation. The second patient had a small nonenhancing midbrain tumor (P12) and was in treatment with chemotherapy and radiation. At two time points during treatment, 2HG was undetectable for 16.5 months. Glycine level was low, and there were no significant changes in T2‐FLAIR images during the period. The third patient presented with a nonenhancing pontine mass (P2), high 2HG (4.9 mM), and normal glycine level by MRS. Following treatment with chemotherapy and radiation, the tumor response, documented by a reduction in T2‐FLAIR hyperintensity, had significant changes in 2HG, tCr, and tNAA levels toward normal when estimated with reference to water (Figure S5). Taken together, these longitudinal cases demonstrate estimates of 2HG and glycine that reflect the clinical state of the tumor, namely, stable levels when the tumor is stable and decreasing levels in response to treatment.

Comparison of MRS with follow‐up clinical MRI in four patients demonstrated an association between high glycine and tumor progression (Figure 4B). In two patients with low glycine (P8 and P11), the tumor responded to treatment and, as a result, the T2‐FLAIR and/or T1‐Gd enhancement volumes were smaller. In contrast, two brainstem tumors (P17 and P19), having undetectable 2HG but high glycine, showed rapid progression after the MRS scans, clinically consistent with a presumptive diagnosis of high‐grade IDH wildtype glioma. A patient with 3.4‐mM glycine (P17) in a gadolinium‐enhancing tumor mass showed increases in the T2‐FLAIR and enhancement volumes in 2 months. Similarly, a patient with a nonenhancing tumor (P19), who presented with very high glycine (4.9 mM) and undetectable 2HG, experienced steadily worsening clinical symptoms and gradual appearance of T1‐Gd enhancement despite aggressive treatment.

**FIGURE 5:**
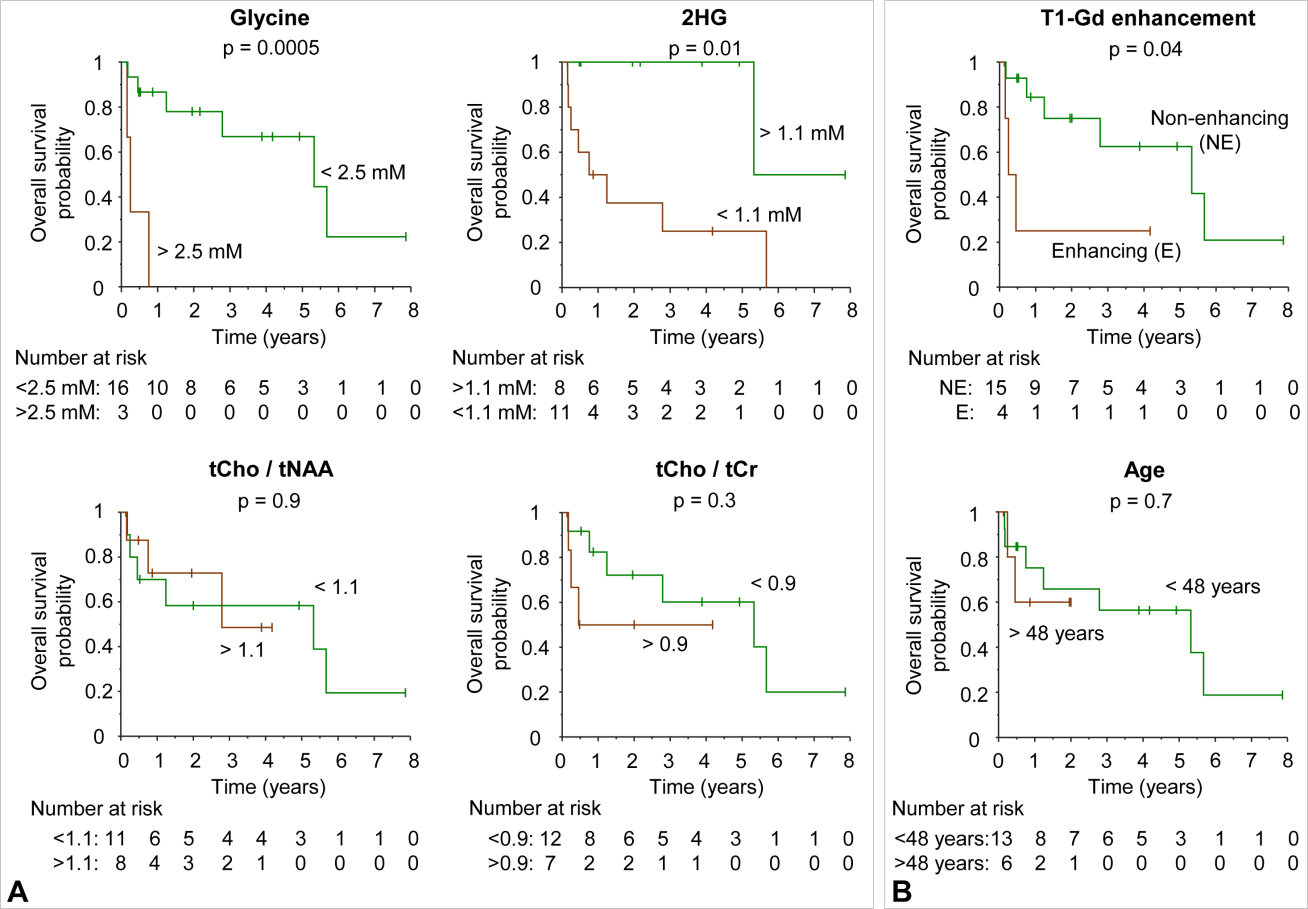
Kaplan–Meier survival analyses of metabolite estimates in 19 brainstem tumor patients. The time along the *x* axis represents the time since the MRS scan date. For subjects who had MRS at multiple time points, the first MRS scan date was used. The *p* value represents the statistical significance of the difference in overall survival between two patient groups stratified by a cutoff value.

Figure 5A presents Kaplan–Meier survival analyses with MRS estimates of metabolites in 19 patients. Glycine demonstrated a strong association with patient survival. The overall survival was significantly shorter in patients with glycine concentrations higher than 2.3 mM compared to those with glycine concentrations lower than 2.3 mM (*p* = 0.0005). The hazard ratio of the high‐glycine tumors relative to the low‐glycine tumors was as high as 7.6. There was also a higher risk of death associated with low concentrations of 2HG. Patients with 2HG levels lower than 1.0 mM showed significantly shorter survival times than those with higher 2HG levels. There was no significant association of tCho/tNAA or tCho/tCr ratio with patient survival [30, 31, 32]. Enhancing tumors were associated with significantly shorter survival compared to nonenhancing tumors (*p* = 0.04), with a hazard ratio lower than that of glycine (3.8 vs. 7.6) (Figure 5B). There was no significant association observed between patient age and overall survival. In addition, there was no significant association of myo‐inositol or glycine + myo‐inositol estimates with patient survival (Figure S6).

## 4 Discussion

We report measurable 2HG in 42% of patients in this study, consistent with a prior study of 10 brainstem tumor patients who had tissue confirmation of IDH in four patients [33]. This study builds on our prior 2HG MRS studies that established sensitivity and specificity of 2HG MRS in nonbrainstem tumors and reliability of the measurement in different areas of the brain and in serial measurements over the range of clinical scenarios [29]. Although metabolite estimation depends on many assumptions that are used in calculating the concentrations, tumors with 2HG > 1.0 mM in the present study can be presumed to harbor an IDH1 or IDH2 mutation, and patients with 2HG < 1.0 mM may be IDH wildtype or could have low cellularity IDH‐mutant tumor. The latter would be false negatives, thus underestimating the percentage of patients with IDH‐mutant tumors. This is of major significance because, in current practice, without a tissue diagnosis of IDH1 or IDH2, the patient does not have access to the FDA‐approved IDH1/2 inhibitor, Vorasidenib. However, establishing 2HG as a reliable biomarker for an IDH mutation could be used as a surrogate in brainstem tumor patients to enable access to the drug.

The presence of high glycine on MRS may indicate a faster growing tumor. In this study, brainstem tumors with elevated glycine progressed rapidly, demonstrating increases in T2‐FLAIR and T1‐Gd enhancement volumes in the months following the MRS exam. Glycine plays a critical role in tumor proliferation. Serine, produced from 3‐phosphoglycerate in the glycolytic pathway, is converted to glycine via serine hydromethyltransferase 1 or 2. The product, glycine, may be generated in either the cytosol or the mitochondria. The critical enzymes in this pathway are amplified in numerous cancers, presumably due to the requirement for macromolecule synthesis via the folate and methionine cycles [10, 11, 12]. These conclusions are consistent with our results. Glycine levels higher than 2.5 mM were associated with shorter overall survival in the present study and a prior survival analysis in supratentorial glioma patients [23]. This suggests that glycine may be a strong predictor of survival and an important prognostic biomarker. Notably, high glycine levels in a nonenhancing tumor can be predictive of developing T1‐Gd enhancement, such as in the patient 19 (P19) in the present study where T1‐Gd enhancement occurred 3 months after the MRS exam and the enhancement volume steadily increased thereafter. There was no significant association of tCho/tNAA or tCho/tCr ratio with patient survival in our data, in contrast with prior studies [30, 31, 32]. Higher tCho in healthy brainstem compared to cerebral regions may be due to the high density of acetylcholine‐releasing cholinergic neurons in the brainstem [34].

Technical challenges associated with MRS measurements of 2HG and glycine were addressed as follows. First, although T2 signal loss is substantial in TE 97‐ms PRESS, this long‐TE MRS outperforms short‐TE MRS because the metabolite signals are better resolved in the long‐TE spectra with attenuated macromolecular signals [23, 24]. The major peak of 2HG at 2.25 ppm is temporally maximum and narrower at TE of 97 ms compared to short TEs (< 30 ms) [6]. The intensity and pattern of the myo‐inositol signal are favorably altered at the 97‐ms TE, allowing for discrimination between glycine and myo‐inositol signals [23]. Second, *B*_1_ calibration was performed on the MRS voxel, which helps to attain more accurate flip angles compared to slice‐based *B*_1_ calibration. Third, time‐domain signals were recorded in multiple blocks, and subsequently, frequency/phase correction was performed in individual spectra prior to final averaging, which is particularly important for improving the signal specificity of weak resonances. Fourth, the basis signals used for spectral fitting, which were prepared with density‐matrix simulations incorporating the actual MRS sequence parameters (i.e., slice‐selective RF and gradient pulse waveforms) [6], were nearly identical to the experimental signals [24]. Fifth, spectral overlap of the 2HG and lipid resonances at 2.25 ppm was addressed using a customized lipid basis set in spectral fitting, in which the lipid 2.25‐ppm signal was predetermined with reference to the strong 0.9‐ppm lipid resonance [26]. Sixth, because all metabolite levels are altered in brain tumors, we quantified metabolites with reference to water, whose contents in brain tumors may be about 20% higher than in normal brain tissue [26]. The water signal was recorded with long TR and short TE to minimize the effects of water T1 and T2 relaxations, which are very different between brain tumors and normal brain. Lastly, every effort was made to position a voxel completely within the lesion (e.g., T2‐FLAIR hyperintensity) to minimize partial volume effects.

A limitation of the present study is the small sample size, which was partially due to the low incidence of brainstem tumors in adults. However, as in patient P4 who was followed serially for more than 3 years with stable, IDH‐detectable nonenhancing disease, MRS offers an opportunity to assess 2HG, make the presumptive diagnosis, and give prognosis. Development of a tumor MRS protocol on clinical scanners, together with a user‐friendly data processing pipeline, will allow for an extended study with a larger number of patients. In patients who are safe to biopsy, increased numbers of confirmed IDH‐mutants in 2HG positive tumors will encourage the use of the biomarker in clinical decision making. Our millimolar estimates of metabolites in tumors with reference to water at 48 M may have uncertainty depending on the actual water concentration, which may vary in the tumors (coefficient of variation of 8% [26]). The water T2 signal loss during the 14‐ms TE was assumed to be negligible. In addition, biopsy confirmation of IDH‐mutation status was relatively low (3/19). The potential of MRS to reliably and noninvasively detect IDH status will be further realized if the MRS results can be confirmed with biopsy analysis during this initial adoption period.

## 5 Conclusion

The present study demonstrates that TE 97‐ms PRESS reliably detects both 2HG and glycine in brainstem tumors. Both 2HG and glycine measures offer predictive insights into the clinical outcome. Noninvasive assessment of the IDH mutation status in brainstem tumors could have significant treatment implications, particularly because treatment with Vorasidenib leads to significant prolongation of progression‐free survival and time to next intervention [35]. Because of the toxicity of radiation to the brainstem, prolonging the time until radiation is needed would be a major clinical benefit. Furthermore, the presence of high glycine on MRS may serve as a biomarker of brainstem tumor aggressiveness with potential transformation to high‐grade disease when it rises or establishes a rapid course when it is high at initial diagnosis. In contrast, low glycine associated with stable 2HG on serial imaging could be a marker of low‐grade stable disease. To date, no such biomarkers exist, and both 2HG and glycine by MRS could be incorporated into initial diagnosis as well as serve as an indicator of stable or impending progression when measured over time. For patients with brainstem gliomas for whom biopsy carries a high risk of neurological impairment, MRS can provide a path to effective treatment in close to 50% of patients who would otherwise undergo radiation therapy early in the disease.

## Author Contributions

**Vivek Tiwari:** writing – original draft, investigation, methodology, software, formal analysis, data curation, visualization. **Sandeep K. Ganji:** writing – review and editing, methodology, software, data curation, visualization. **Zhongxu An:** writing – review and editing, methodology, software, data curation, visualization. **Marco C. Pinho:** writing – review and editing, validation, project administration. **Larry T. Davis:** writing – review and editing, validation, project administration. **Colin D. McKnight:** writing – review and editing, validation, project administration. **Julia D. Berry:** writing – review and editing, validation, project administration. **Bret C. Mobley:** writing – review and editing, validation, project administration. **Leo Y. Luo:** writing – review and editing, validation, project administration. **Alexander C. Mohler:** writing – review and editing, validation, project administration. **Ryan T. Merrell:** writing – review and editing, validation, project administration. **Patrick D. Kelly:** writing – review and editing, validation, project administration. **Reid C. Thompson:** writing – review and editing, validation, project administration. **Bruce E. Mickey:** writing – review and editing, validation, project administration. **Craig R. Malloy:** writing – review and editing, validation, project administration. **John C. Gore:** writing – review and editing, resources, project administration, funding acquisition. **Toral R. Patel:** writing – review and editing, conceptualization, validation, project administration. **Elizabeth A. Maher:** writing – review and editing, conceptualization, validation, formal analysis, supervision, project administration, funding acquisition. **Changho Choi:** writing – original draft, conceptualization, methodology, software, formal analysis, visualization, supervision, funding acquisition.

## Conflicts of Interest

S.K.G. is an employee of Philips.

## Supporting information


**Table S1:** Minimum reporting standards.
**Figure S1:** In vivo TE 97 ms PRESS spectra from the entire 20 brainstem tumor subjects enrolled in the present study (P1—P20) are presented together with the spectral fitting results and voxel positioning in T2‐FLAIR images. The data from P20 was excluded in subsequent analysis because of the low signal‐to‐noise ratio. Each spectrum was normalized to the unsuppressed water signal from the voxel acquired with 20 s TR and 14 ms TE. Shown on the left in individual subjects are patient number, patient's age in years, and gender (Male or Female). The numbers shown on the right represent, top to bottom, the FWHM of the tCho singlet, voxel size, signal average number, and the background noise level in institutional unit. Here the tCho FWHM was measured from the LCModel‐returned total choline signal (GPC + PCh). The background noise level was calculated as the standard deviation of the LCModel‐returned residuals between 0.5–4.0 ppm. For P1—P19 spectra, the mean values of tCho FWHM, voxel size, signal averaging, and background noise level were 7.3 ± 1.5 Hz (range 5.2–10.3 Hz), 4.9 ± 2.0 mM (range 1.5–8 mL), 381 ± 210 (range 128–896), and 71 ± 23 (range 39–136), respectively. The P20 data, whose background noise level was higher than our cutoff value of 150, was excluded in subsequent analysis.
**Figure S2:** (**A**) The number of PRESS signal averages is plotted vs. voxel size for the 20 patients of the present study. (**B**) The tCho (total choline) SNR (singlet height to noise ratio) is plotted vs. voxel size for the 20 patients of the present study. The noise level was calculated as the standard deviation of the LCModel‐returned residuals between 0.2–4.0 ppm. The case of patient 20 (P20 in Figure S1), which was excluded in subsequent analysis due to low SNR, is indicated by red X in the figures.
**Figure S3:** The spectra in Figure 1 are presented with LCModel‐returned signals of 2HG, GABA, glutamate (Glu), glutamine (Gln), glycine, and myo‐inositol (mI) and their millimolar estimates and CRLBs.
**Figure S4:** Eleven spectra from patient 4 (P4), who had follow‐up MRS scans over 3.3 years, are shown together with spectral fitting results. The scan time points relative to the first MRS exam are shown for individual scans. The voxel size was 7.4 ± 1.0 mL (range 5.76–8 mL) and the number of signal averages was 267 ± 25 (range 256–320). The mean linewidth of the tCho singlet was 7.5 ± 1.0 Hz (range 6.1–9.7 Hz). The ratio of the LCModel‐returned tCho peak amplitude with respect to the LCModel‐returned residuals was 85 ± 9 (range 64–97).
**Figure S5:** (**A**) The voxel positionings in MRS exams in Patient 2 (P2) at two time points (0 and 13 months) are shown on T2‐FLAIR images (voxel in red and shimming volume in yellow). The patient had chemoradiation treatment between the baseline and follow‐up MRS exams. (**B**) The TE 97 ms PRESS spectra obtained at the two time points (TR = 2 s and 128 averages) are presented together with LCModel outputs. The spectra were normalized to the water signal obtained with (TR, TE) of (20 s, 14 ms). (**C**) The LCModel estimates of five‐metabolite signals of the MRS at 0 and 13 months, normalized to water, are presented for three water acquisitions, (TR, TE) = (20 s, 14 ms), (2 s, 14 ms), and (2 s, 97 ms). The metabolite‐to‐water ratios of the baseline and follow‐up exams were normalized to the baseline ratio for each metabolite.
**Figure S6:** (Upper panel) The estimates of myo‐inositol and glycine + myo‐inositol are bar graphed together with the glycine estimates in the same order of the 19 patients. (Lower panel) Kaplan–Meier analyses of myo‐inositol and glycine + myo‐inositol.

## Data Availability

The data that support the findings of this study are available on request from the corresponding author. The data are not publicly available due to privacy or ethical restrictions.
