# Supplementary material for: Magnetic Resonance Spectroscopy of 2‐Hydroxyglutarate and Glycine in Adult Subjects With Brainstem Gliomas
Source: NMR Biomed. 2025 Sep 30;38(11):e70153. doi: 10.1002/nbm.70153 (PMC12481179; doi:10.1002/nbm.70153)

**SUPPORTING INFORMATION**

**Table S1**. Minimum reporting standards.

| Field strength | 3T |
| --- | --- |
| Manufacturer, Model | Philips Achieva, Elition |
| Proton RF coils | Body transmit coil and 32-channel receive coil |
| Pulse sequence | PRESS, STEAM |
| Volume of interest (VOI) locations | Brainstem |
| Nominal VOI size | 1.5 - 8 mL |
| Repetition time (TR) and echo time (TE) | TR 2 s, and TE 97 ms (TE1 = 32 ms and TE2 = 65 ms) for water suppressed PRESS. TR 20 s, TE 14 ms, and TM 19 ms for unsuppressed STEAM water acquisition. |
| Total number of excitations or acquisitions per spectrum | 128 - 896 |
| Water suppression method | Four-pulse variable flip angle scheme |
| Shimming method | Vendor-supplied method (Pencil beam) |
| Analysis software | LCModel |
| Processing steps deviating from quoted reference or product | sptype = ‘tumor’ |
| Output measure | Absolute concentration |
| Quantification references | Brain white-matter total creatine at 6 mM |
| Reported variables (SNR, linewidth (with reference peaks)) | SNR, FWHM |
| Data exclusion criteria | FWHM < 10 Hz; Extensive baseline distortion; Noise level in institutional unit < 150 (see Figure S1); |
| Quality measures of postprocessing model fitting (eg, CRLB, goodness of fit, SD of residual) | Goodness of fit and SD of residuals |
| Sample spectrum | Figure 1 |

**Figure S1**. In vivo TE 97 ms PRESS spectra from the entire 20 brainstem tumor subjects enrolled in the present study (P1 - P20) are presented together with the spectral fitting results and voxel positioning in T2-FLAIR images. The data from P20 was excluded in subsequent analysis because of the low signal-to-noise ratio. Each spectrum was normalized to the unsuppressed water signal from the voxel acquired with 20 s TR and 14 ms TE. Shown on the left in individual subjects are patient number, patient’s age in years, and gender (Male or Female). The numbers shown on the right represent, top to bottom, the FWHM of the tCho singlet, voxel size, signal average number, and the background noise level in institutional unit. Here the tCho FWHM was measured from the LCModel-returned total choline signal (GPC + PCh). The background noise level was calculated as the standard deviation of the LCModel-returned residuals between 0.5 - 4.0 ppm. For P1 - P19 spectra, the mean values of tCho FWHM, voxel size, signal averaging, and background noise level were 7.3±1.5 Hz (range 5.2 - 10.3 Hz), 4.9±2.0 mM (range 1.5 - 8 mL), 381±210 (range 128 - 896), and 71±23 (range 39 - 136), respectively. The P20 data, whose background noise level was higher than our cutoff value of 150, was excluded in subsequent analysis.


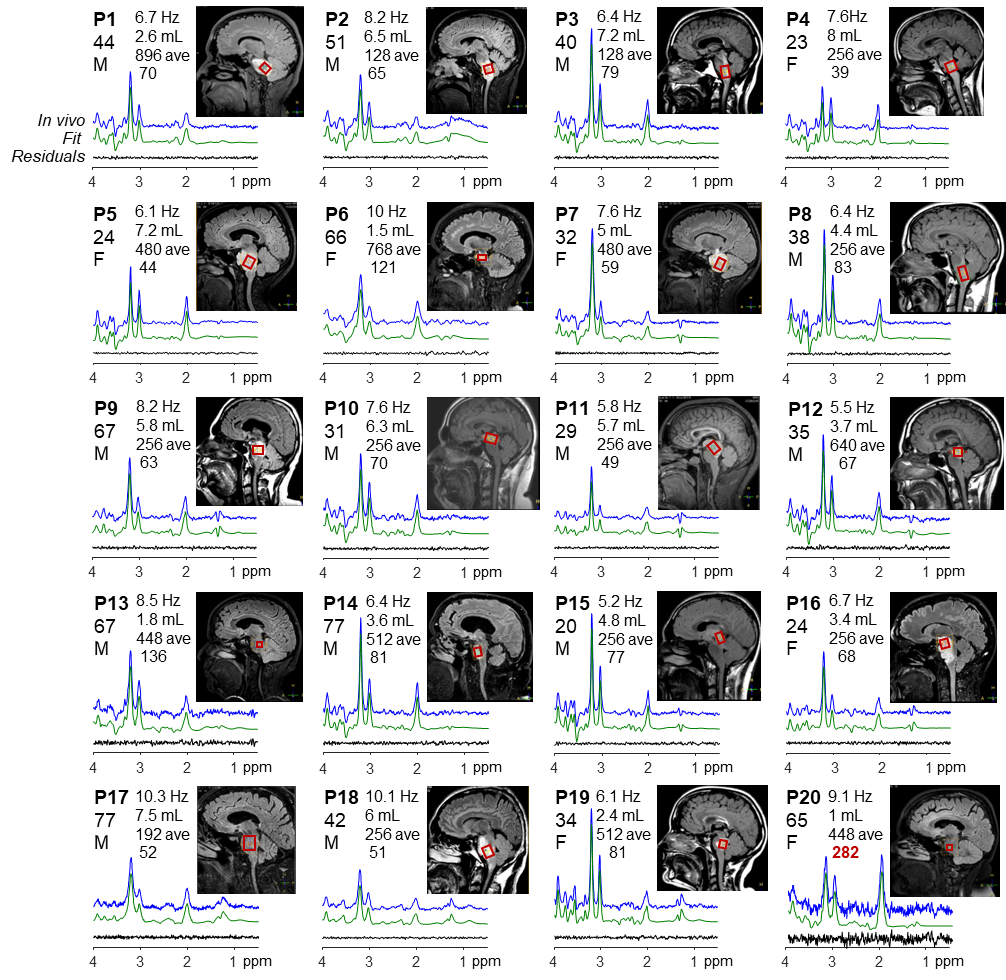


**Figure S2**. (**A**) The number of PRESS signal averages is plotted vs voxel size for the 20 patients of the present study. (**B**) The tCho (total choline) SNR (singlet height to noise ratio) is plotted vs voxel size for the 20 patients of the present study. The noise level was calculated as the standard deviation of the LCModel-returned residuals between 0.2 - 4.0 ppm. The case of patient 20 (P20 in Figure S1), which was excluded in subsequent analysis due to low SNR, is indicated by red X in the figures.


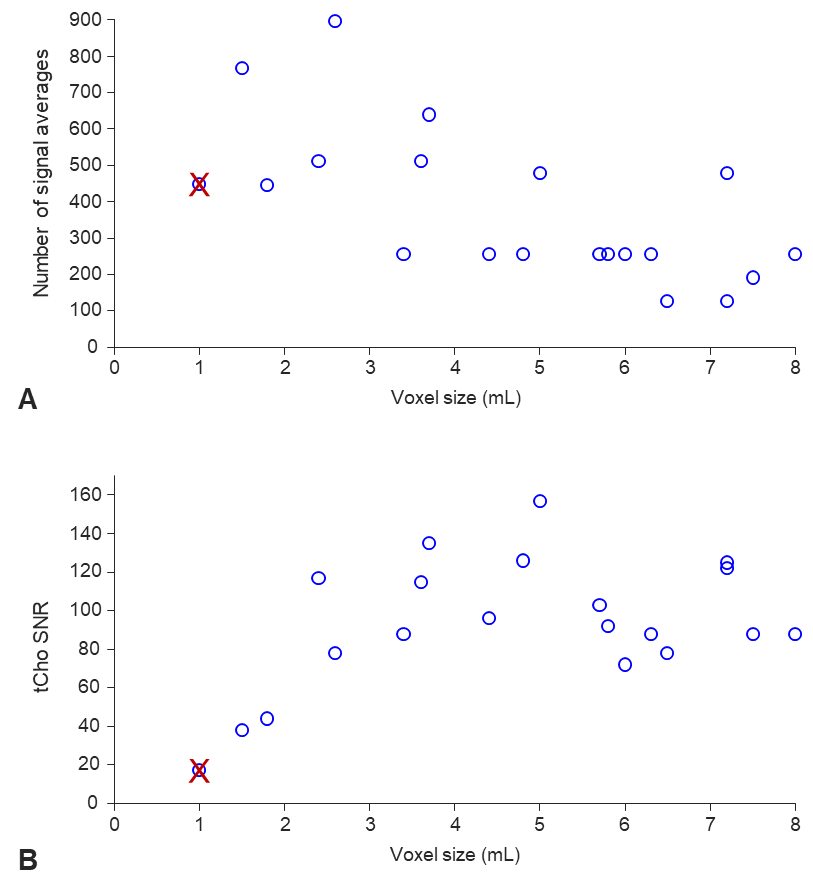


**Figure S3**. The spectra in Figure 1 are presented with LCModel-returned signals of 2HG, GABA, glutamate (Glu), glutamine (Gln), glycine, and myo-inositol (mI) and their millimolar estimates and CRLBs.


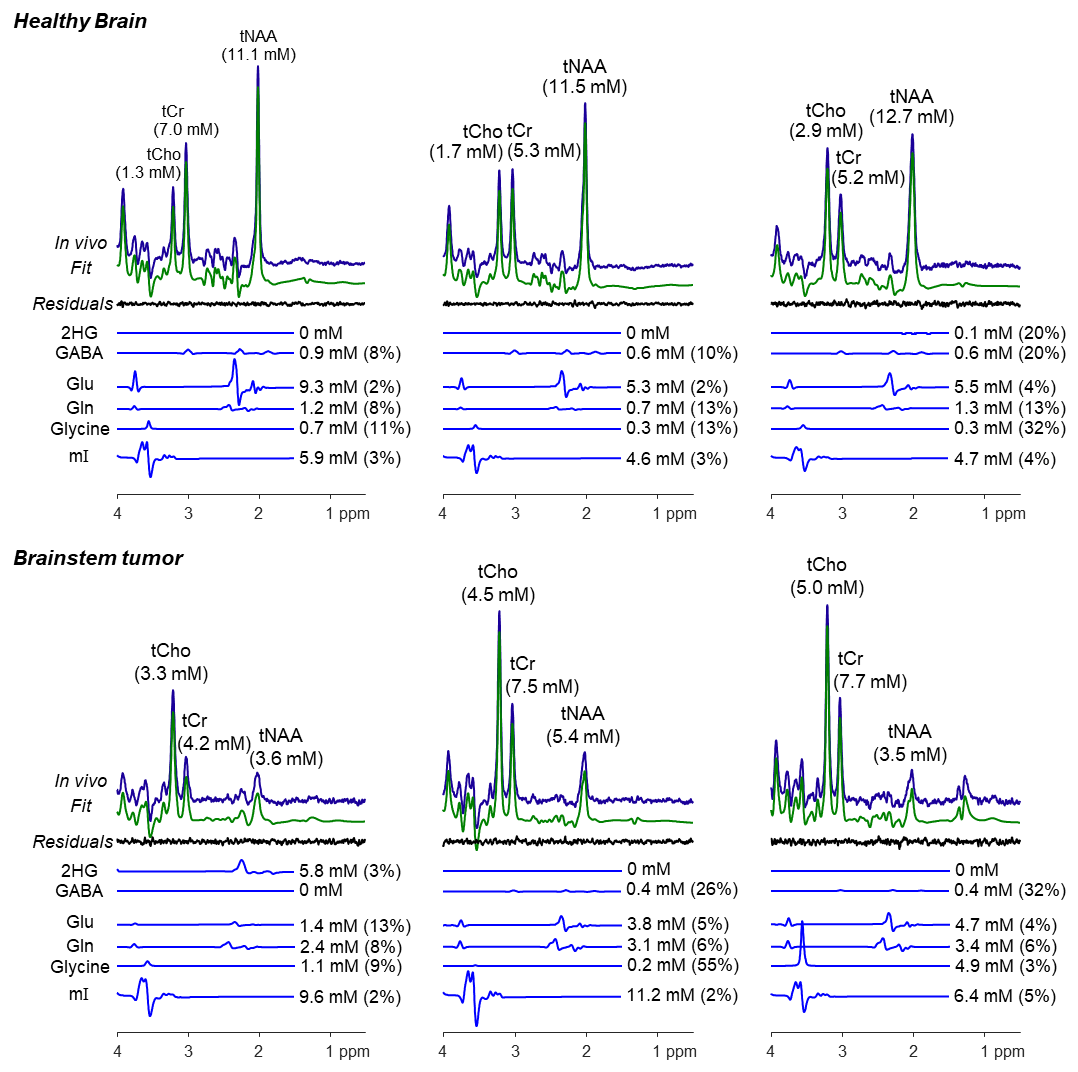


**Figure S4**. Eleven spectra from patient 4 (P4), who had follow-up MRS scans over 3.3 years, are shown together with spectral fitting results. The scan time points relative to the first MRS exam are shown for individual scans. The voxel size was 7.4±1.0 mL (range 5.76 - 8 mL) and the number of signal averages was 267±25 (range 256 - 320). The mean linewidth of the tCho singlet was 7.5±1.0 Hz (range 6.1 - 9.7 Hz). The ratio of the LCModel-returned tCho peak amplitude with respect to the LCModel-returned residuals was 85±9 (range 64 - 97).


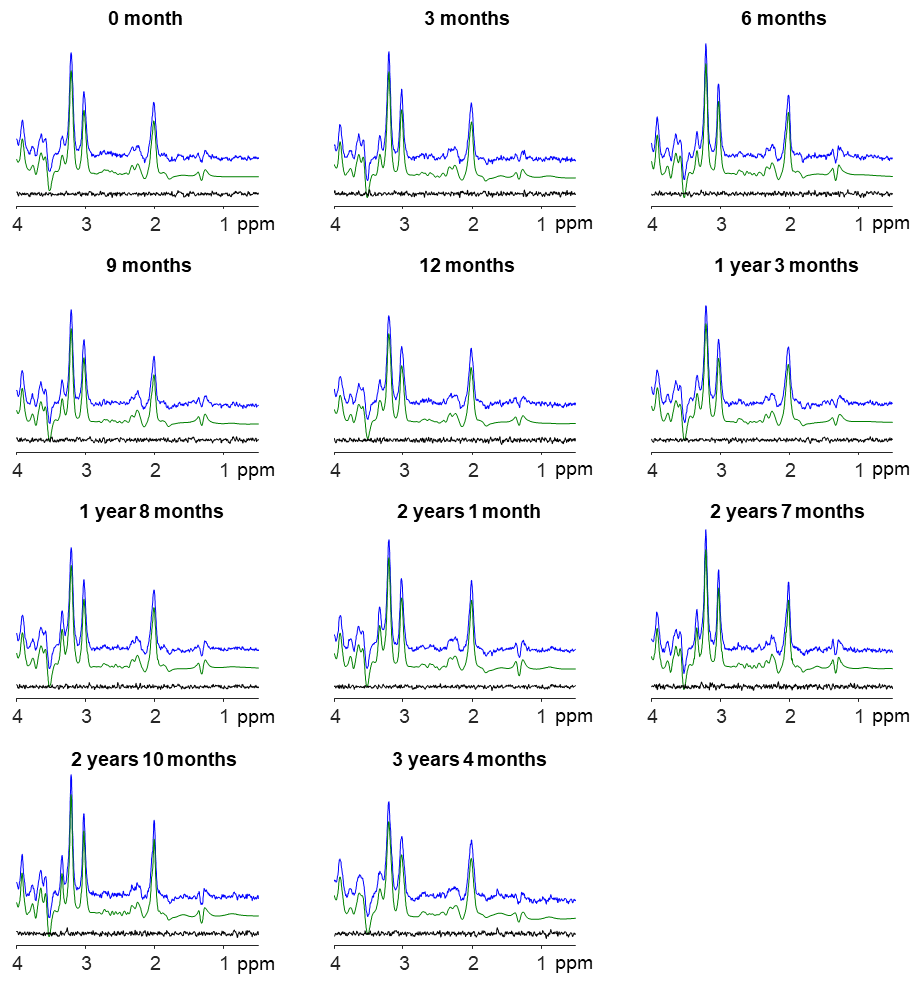


**Figure S5**. (**A**) The voxel positionings in MRS exams in Patient 2 (P2) at two time points (0 and 13 months) are shown on T2-FLAIR images (voxel in red and shimming volume in yellow). The patient had chemoradiation treatment between the baseline and follow-up MRS exams. (**B**) The TE 97 ms PRESS spectra obtained at the two time points (TR = 2 s and 128 averages) are presented together with LCModel outputs. The spectra were normalized to the water signal obtained with (TR, TE) of (20 s, 14 ms). (**C**) The LCModel estimates of five-metabolite signals of the MRS at 0 and 13 months, normalized to water, are presented for three water acquisitions, (TR, TE) = (20 s, 14 ms), (2 s, 14 ms), and (2 s, 97 ms). The metabolite-to-water ratios of the baseline and follow-up exams were normalized to the baseline ratio for each metabolite.


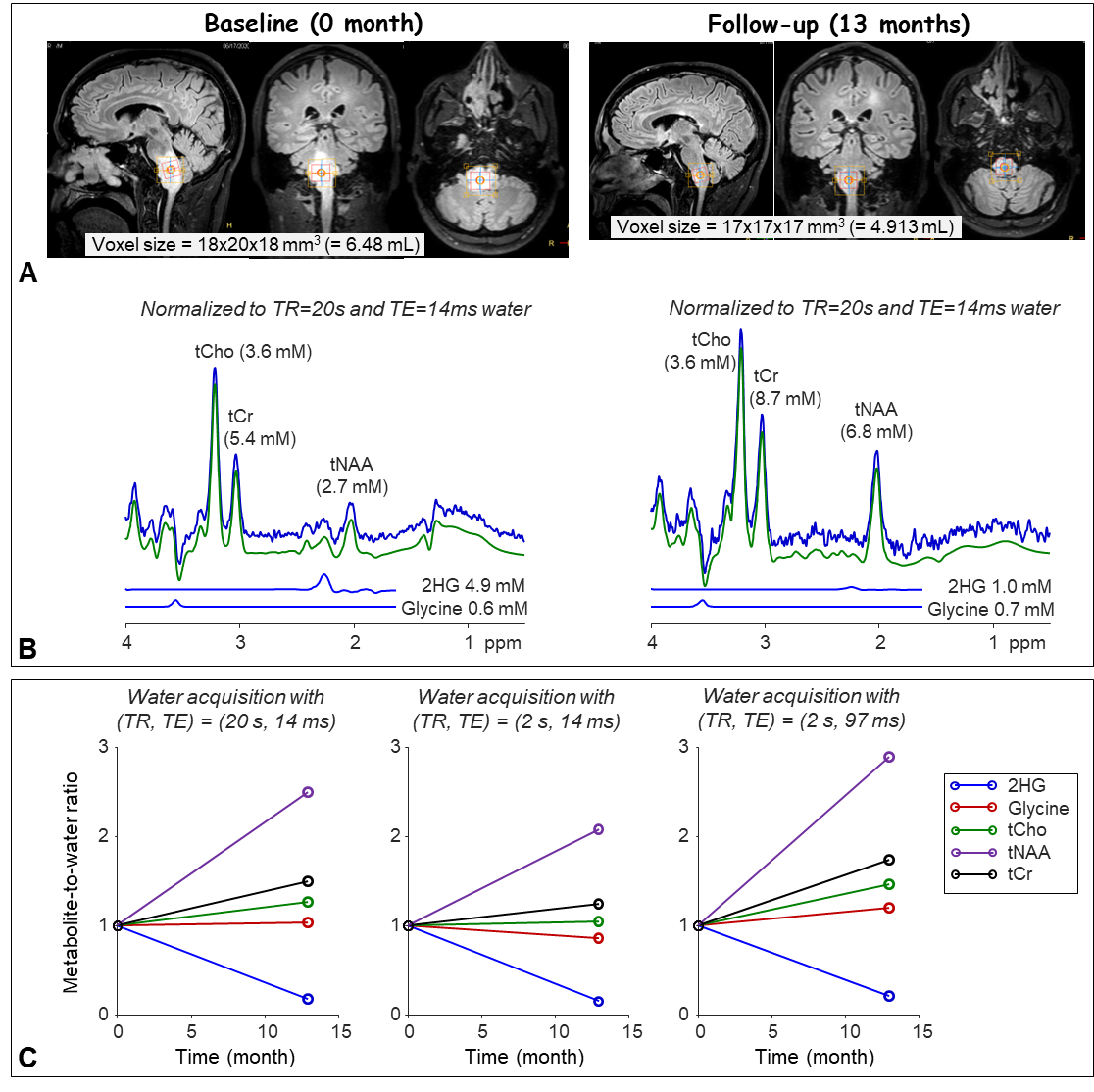


**Figure S6**. (Upper panel) The estimates of myo-inositol and glycine + myo-inositol are bar graphed together with the glycine estimates in the same order of the 19 patients. (Lower panel) Kaplan-Meier analyses of myo-inositol and glycine + myo-inositol.


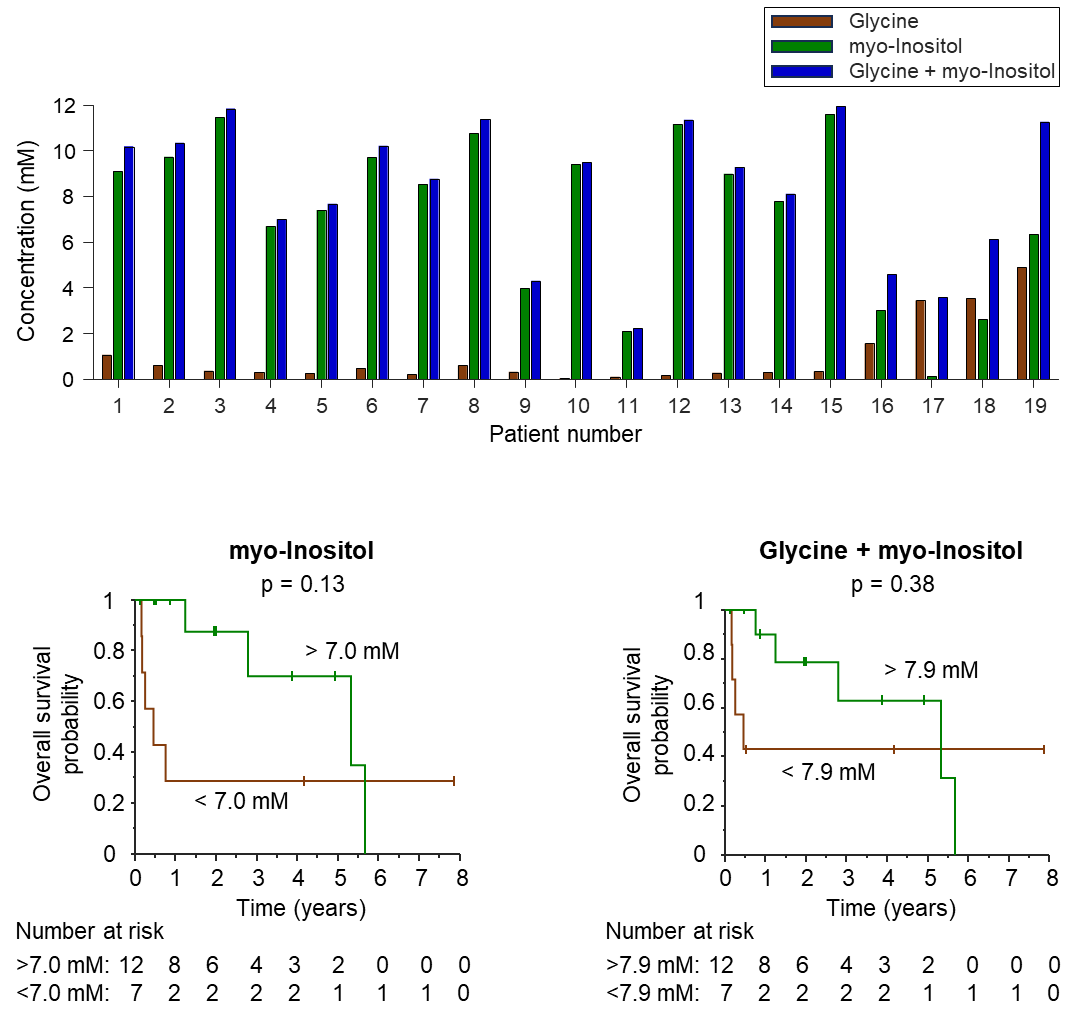

Supplement: Supplementary file 1 — Table S1: Minimum reporting standards. Figure S1: In vivo TE 97 ms PRESS spectra from the entire 20 brainstem tumor subjects enrolled in the present study (P1—P20) are presented together with the spectral fitting results and voxel positioning in T2‐FLAIR images. The data from P20 was excluded in subsequent analysis because of the low signal‐to‐noise ratio. Each spectrum was normalized to the unsuppressed water signal from the voxel acquired with 20 s TR and 14 ms TE. Shown on the left in individual subjects are patient number, patient's age in years, and gender (Male or Female). The numbers shown on the right represent, top to bottom, the FWHM of the tCho singlet, voxel size, signal average number, and the background noise level in institutional unit. Here the tCho FWHM was measured from the LCModel‐returned total choline signal (GPC + PCh). The background noise level was calculated as the standard deviation of the LCModel‐returned residuals between 0.5–4.0 ppm. For P1—P19 spectra, the mean values of tCho FWHM, voxel size, signal averaging, and background noise level were 7.3 ± 1.5 Hz (range 5.2–10.3 Hz), 4.9 ± 2.0 mM (range 1.5–8 mL), 381 ± 210 (range 128–896), and 71 ± 23 (range 39–136), respectively. The P20 data, whose background noise level was higher than our cutoff value of 150, was excluded in subsequent analysis. Figure S2: (A) The number of PRESS signal averages is plotted vs. voxel size for the 20 patients of the present study. (B) The tCho (total choline) SNR (singlet height to noise ratio) is plotted vs. voxel size for the 20 patients of the present study. The noise level was calculated as the standard deviation of the LCModel‐returned residuals between 0.2–4.0 ppm. The case of patient 20 (P20 in Figure S1), which was excluded in subsequent analysis due to low SNR, is indicated by red X in the figures. Figure S3: The spectra in Figure 1 are presented with LCModel‐returned signals of 2HG, GABA, glutamate (Glu), glutamine (Gln), glycine, and myo‐inositol [file NBM-38-e70153-s001.docx]
